# The evidence on tiotropium bromide in asthma: from the rationale to the bedside

**DOI:** 10.1186/s40248-017-0094-3

**Published:** 2017-05-04

**Authors:** Dejan Radovanovic, Pierachille Santus, Francesco Blasi, Marco Mantero

**Affiliations:** 1 Department of Biomedical and Clinical Sciences (DIBIC), University of Milan, Pulmonary Unit, Ospedale L. Sacco, ASST Fatebenfratelli-Sacco, Milan, Italy; 2 Department of Pathophysiology and Transplantation, University of Milan, Cardio-thoracic unit and Adult Cystic Fibrosis Center, Fondazione IRCCS Ca’ Granda Ospedale Maggiore Policlinico, Milan, Italy

**Keywords:** Airway remodeling, Anticholinergic, Asthma, Exacerbation, Forced expiratory flow, Inflammation, Muscarinic receptor, Poor control, Tiotropium

## Abstract

Severe and poorly controlled asthma still accounts for a great portion of the patients affected. Disease control and future risk management have been identified by international guidelines as the main goals in patients with asthma. The need for new treatment approaches has led to reconsider anticholinergic drugs as an option for asthma treatment. Tiotropium is the first anticholinergic drug that has been approved for children and adults with poorly controlled asthma and is currently considered as an option for steps 4 and 5 of the Global Initiative for Asthma. In large randomized clinical trials enrolling patients with moderate to severe asthma, add-on therapy with tiotropium has demonstrated to be efficacious in improving lung function, decreasing risk of exacerbation and slowing the worsening of disease; accordingly, tiotropium demonstrated to be non inferior compared to long acting beta-agonists in the maintenance treatment along with medium to high inhaled corticosteroids. In view of the numerous ancillary effects acting on inflammation, airway remodeling, mucus production and cough reflex, along with the good safety profile and the broad spectrum of efficacy demonstrated in different disease phenotypes, tiotropium can represent a beneficial alternative in the therapeutic management of poorly controlled asthma. The present extensive narrative review presents the pharmacological and pathophysiological basis that guided the rationale for the introduction of tiotropium in asthma treatment algorithm, with a particular focus on its conventional and unconventional effects; finally, data on tiotropium efficacy and safety. from recent randomized clinical trials performed in all age categories will be extensively discussed.

## Background

Asthma is a chronic inflammatory disease of the airways characterized by a complex pathophysiology and caused by different etiological factors which contribute to the heterogeneity of clinical presentation and to the severity of disease. Asthma can manifest both in childhood and in elderly patients [1], the number of patients affected worldwide being estimated in 235 million [2], ranging from 1 to 18% of the population in different countries [3]. Asthma is characterized by a variable combination of symptoms such as wheeze, cough, shortness of breath, chest tightness and by different degrees of airflow obstruction and airway hyper-responsiveness that can vary in time and intensity [3, 4]. The worsening of respiratory symptoms and thus disease exacerbations can be caused by direct or mediated mechanical, physical and infectious stresses that increase bronchial inflammation and cause an acute worsening of airflow obstruction [3]. The latter circumstance, especially in elderly patients, can be complicated by comorbid conditions requiring a multidisciplinary therapeutic approach [5, 6]. Poor control of symptoms and acute exacerbations requiring emergency room admittance and hospitalization expose patients to a poor quality of life, high mobility and a significant economic burden on the healthcare system and society [7]. Although to a minor extent compared with chronic obstructive pulmonary disease (COPD) [8, 9], severe and poorly controlled asthma still accounts for a significant in-hospital mortality, especially in children, elderly and mechanically ventilated patients [10, 11], or patients from low income countries [2]. For many years, important efforts have been spent to optimize asthma management; however, despite the availability of new treatment options [12], international surveys continue to provide evidence for suboptimal asthma control in many countries [13], with poor control of symptoms and exposure to risk of exacerbation in some cases affecting more than 50% of patients [14, 15]. The need for new treatment approaches has led to reconsider anticholinergic drugs as a pharmacological treatment option for asthma [16]. While also other muscarinic antagonists are currently being considered for approval in asthma [16], the only anticholinergic drug introduced so far in the treatment algorithms of major international guidelines is represented by tiotropium bromide. In the last few years numerous clinical trials proved the efficacy of tiotropium both in adult and younger patients for the chronic treatment of asthma. Due to the numerous non-bronchodilator properties expressed by tiotropium, its efficacy may not completely rely on the reduction of the airways’ cholinergic bronchomotor tone, but also on anti-inflammatory and modulatory effects of the structures involved in the complex molecular and cellular pathophysiology of asthma. The aim of the present extensive narrative review is to highlight the pharmacological and non-bronchodilator properties of tiotropium and to present the data of clinical trials conducted so far to examine its role in connection with the current pharmacological treatment paradigm in patients with asthma.

### The rationale for anticholinergic drugs in asthma

#### The cholinergic system in human airways

The innervation of human airways is mainly represented by the parasympathetic system [17]. The sympathetic system is less represented, however, some sympathetic endings can be found on ganglion cell bodies and, therefore, may be involved in the modulation of cholinergic traffic [18, 19]. Acetylcholine (ACh) is the major neurotransmitter of the parasympathetic nervous system and acts both at the ganglionic transmission and the neuroeffector junctions [16, 18]. ACh has two classes of receptors: nicotinic and muscarinic. The first are ligand-gated ion channels, mainly present on the peribronchial ganglion; muscarinic receptors are G-protein-coupled receptors and more widely distributed in the lung [19]. Bronchoconstriction is primarily regulated by muscarinic receptors (M_R_); of the five receptor subtypes, M_1_, M_2_ and M_3_ are expressed in the lung and in the bronchial tree. M_1R_ are mainly distributed in the peripheral lung tissue and in the alveolar walls [16] within parasympathetic ganglia and regulate cholinergic transmission [20]. M_2R_ are found in post-ganglionic nerves where they serve as auto-receptors, on smooth muscle cells (SM) where they are coupled to inhibitory G-proteins [16, 21] and on fibroblasts [16]. Together with M_2R_, M_3R_ are the most represented in human airways; they are predominantly expressed in SM cells and mediate SM ACh-induced contraction [16, 20]. In central airways this mechanism is mediated by vagal innervation, in the periphery of the lung M_3R_ function is mediated by ACh released in response to inflammatory stimuli from epithelial cells expressing choline acetyl-transferase (ChAT) [16]. M_3R_ serve also as mediators of airways blood vessels and can be found in sub-mucosal glands where they are responsible for mucus secretion [22].

Although in normal conditions adrenergic sympathetic, cholinergic and non-cholinergic parasympathetic nerves are all active, vagal innervation is considered the major determinant of airways’ tone [22] and is regarded as the major reversible pathophysiological mechanism responsible for airflow obstruction in COPD. In asthma, an up-regulated release of ACh causes an increased bronchial tone, bronchial hyper-responsiveness and reflex bronchoconstriction [23] which in turn contribute to the narrowing of the airways causing respiratory symptoms and eventually asthma exacerbations. The accurate reason for an increased ACh activity is still unknown, but it has been postulated to derive from an increased susceptibility of the cholinergic system, increased intracellular signaling of muscarinic receptors or by activation of prejunctional facilitatory receptors on cholinergic nerve endings, leading to up-regulated synthesis through higher activity of ChAT or by reduced degradation through choline-esterases [24].

In parallel to the vagal-mediated ACh production, the non-neuronal cholinergic system has an important regulatory role in the homeostasis of airway cells. ACh is responsible for numerous cell vital functions as cell-cycle, proliferation, differentiation, migration and chemokinesis [21]. Increasing evidences suggest that in the airways, non-neuronal ACh can affect several transduction pathways such as G-proteins, phospholipase C, effector kineses, classical and non selective ion and cation channels, and large conductance voltage and calcium-activated potassium channels [21]. Although in some cases cholinergic effects may be produced by mediators other than ACh on M_R_ [21], almost every cell type present in the airways expresses components of the cholinergic system, such as M_R_, ChAT, the high affinity choline transporter (CHT-1), as well as ACh itself [24]. Non-neuronal production of ACh could be particularly relevant in peripheral airways, where cholinergic innervation is scarce. A production of non-neuronal ACh may also be responsible, together with the parasympathetic vagal system, in the modulation of the bronchomotor tone, both in basal conditions and in a disease-state [21]. Given these evidences, the blockade of M_R_ by cholinergic antagonists may not always seem beneficial. The effect will rely mostly on drug’s specific receptor selectivity, kinetics, dynamics and on the molecules the cholinergic antagonists interact with. In asthma, the usual regulatory role of M_2R_ can be overcome by eosinophil release during allergen-challenges, while M_2R_ in some cases can also be dysfunctional [25]. An increased activation of M_3R_ by ACh during airway inflammation states has an effect on SM remodeling, mucus production and the fostering of the inflammatory response [26]. Although it is still not clear if an increased parasympathetic tone is due to the inflammatory state present in asthma or represents a pathophysiological mechanism by itself, the rationale for the application of cholinergic antagonists in asthma appears robust [16, 20] and the non-neuronal cholinergic represents a favorable target.

#### Unmet needs in asthma

To obtain and maintain disease control, until 2015, long acting β2-agonists (LABA) represented the only inhaled bronchodilator treatment in international guidelines, which recommended a stepwise approach according to which LABA in association with inhaled corticosteroids (ICS) at low doses were added beginning from step 3, to be increased in step 4 and 5 [27, 28]. In the last decade the paradigm of asthma control has been evolving along with the increased awareness that any intervention employed to obtain the disease control should consider the management of patients’ future risk [29, 30]. The latter should include, among others, the minimization of adverse events while providing the optimal treatment coverage, the prevention of lung function decline and reduced lung growth in children and, most importantly, the prevention of recurrent exacerbation and especially severe relapses leading to emergency department access and hospitalization [4, 30]. Major risk factors related to future exacerbations are represented by a previous history of exacerbations, increased use of oral corticosteroids and rescue medications, obesity, worse lung function, poor adherence to inhaled therapy, low Asthma Control Questionnaire (ACQ-7) score, co-morbid diseases, chronic sinusitis and cigarette smoke (Table 1) [3, 31–43]. Despite poor inhaler technique has been demonstrated to be related to worse asthma control [44, 45], most of the analyses designed to evaluate risk factors predictive of future exacerbations did not include it in the regressions [37]. The definition of full control in international guidelines includes the use of reliever medication less than twice a week, having no limitation in daily life activities, normal lung function and absence of nocturnal symptoms [3]. Despite this approach, and the numerous pharmacological and clinical interventions now available, there is frequently a misperception of asthma control among patients [40]. In a recent multicenter European study involving more than 8,000 patients, sub-optimal asthma control was found in 56.2% of patients [38], while in a large cohort of patients that followed a guideline-derived asthma control management [15], although the majority of patients treated with LABA/ICS controller therapy achieved well controlled asthma, 29% of patients remained poorly controlled. Moreover, severe asthma exacerbations, often triggered by viral infections, can occur even in patients with mild asthma that usually appears well controlled [46]. Although the role played by poor compliance to inhaled therapy and the little awareness of the disease severity among patients [40] has been largely recognized as a weak point in asthma control, some alternative mechanisms may in part justify this phenomenon.

**Table 1:** Factors associated with increased risk of poor asthma control in adults and the pediatric population

Determinants for poor control	Pediatric	Adults
Clinical and functional		
Exacerbations in the previous year (previous 12 months)	X	X
Hospitalizations in the previous year (previous 12 months)	X	X
Respiratory infections (previous 12 months)	X	
Oral corticosteroid use (previous 12 months)	X	X
SABA prescriptions (1x200 dose canister/month)		X
Healthcare utilization	X	X
Poor lung function		X
Sputum or blood eosinophilia		X
Variability of asthma control	X	X
ACQ-7 < 15	X	X
Demographic		
Female		X
Age (40 to 64 years old)		X
Comorbidities		
GERD		X
Obesity	X	X
Overweight		X
Low birth weight	X	
OSA-sleep disordered breathing	X	X
Allergic rhinitis	X	X
Congestive heart failure		X
Drug exposure		X
Psychological		
Anxiety and depression		X
Misperception of disease	X	X
Low expectations		X
Poor knowledge of disease	X	X
Parent related severity of disease	X	
Patient-independent		
Doctor-related attitudes		X
Patient-dependent		
Weight gain		X
Low adherence		X
Poor inhaler technique		X
Active and past smoking		X
Passive smoking	X	

As for gender-related risk, different authors demonstrated an association with poor asthma control and both female and male gender (mild and severe exacerbations for female gender and higher SABA usage for males). ACQ-7, Asthma control questionnaire; SABA, Short acting β2 agonists; mo, months. Data are from [3, 31–43]

#### Why muscarinic antagonists as add-on therapy in asthma

In the Symbicort Maintenance and Reliever Therapy (SMART) trial, chronic use of LABA has been related to a small but significant increase in risk of asthma-related deaths, especially among African Americans [47], rising the attention of the American Food and Drug Administration (FDA) on the possible role of a step-down approach of LABA in patients treated with LABA/ICS fixed dose combination (FDC), especially in asthmatic children [48–51]. Asthma control, exacerbations [52] and inflammation can worsen adding LABA to ICS [53]. It has been hypothesized that in asthma, long term use of LABA not associated with ICSs, is accompanied by down regulation of β2 adrenoreceptors and thus by tachyphylaxis [54], with a loss of bronchoprotection [55, 56] and a reduction in responsiveness to reliever therapy with salbutamol [57, 58]. A genetic-based sub-sensitivity of response to LABA/ICS FDC treatment can also derive from the Arg16/Arg16 β2-receptor polymorphism, with an increased airway hyper-responsiveness in affected subjects [59–61]. Moreover, the efficacy and potency of β2-agonists is gradually reduced in presence of increasing concentrations of contractile stimuli, including M_R_ agonists and histamine both in vitro [62, 63] and in vivo [64]. The effect can be ascribed to the cross-talk between G_q_-coupled muscarinic M_3R_ or histamine H_1_ receptors and G_s_-coupled β2-adrenoceptors [65–67]. Another pathway inducing β2-adrenoceptor desensitization can be mediated by β2-agonists involving phosphorylation and activation of G-protein-coupled receptor kinase 2 [68]. The down regulation and uncoupling of pre-junctional β2-adrenoreceptors secondary to prolonged LABA stimulation can increase cholinergic transmission [54], which acts via M_3R_ and protein-kinase C phosphorylation. The administration of long acting muscarinic antagonists (LAMA) can thus counteract both the augmented cholinergic transmission and protect against LABA induced sub-sensitivity [69].

In this view, the application of antimuscarinic drugs in asthma had to be expected but was initially largely underestimated [70]; in fact, although they proved to be efficacious both in asthma and COPD especially in the acute setting [71], until a few years ago LAMA and short acting muscarinic antagonists (SAMA) were not part of the long term treatment strategy of international guidelines. This might be attributed to inconsistent results previously reported with SAMA (atropine and ipratropium) [72] that proved to be less effective in terms of acute bronchodilation when compared to salbutamol [72], which could be secondary to the lower drug doses [23] or, more probably, to the poor receptor selectivity of atropine and ipratropium. In fact, both anticholinergic molecules are not selective for M_3R_. An unselective blockade of M_2R_, which usually acts as a pre-synaptic auto-receptor for the blockade of further release of ACh from nerves to SM cells, can overcome the M_3R_ antagonism and thus favor bronchoconstriction [23, 73]. The rationale for the combination of LABA and LAMA in the maintenance therapy in asthma is also favoured by the interaction between M_R_ and β2-adrenoceptors in the airways [74]. In fact, administration of formoterol has been demonstrated to induce an upregulation of M_3R_ [75]. The augmented ACh release secondary to uncoupling of β2-adrenoceptors during prolonged exposure to LABA might be prevented by administration of LAMA, which can also prevent LABA induced sub-sensitivity [54]. The cross-talk between adrenergic and cholinergic receptors might also justify a synergistic action of concomitant administration of LAMA and LABA/ICS therapy, by which LAMA may protect against adverse molecular effects secondary to long exposure to LABA [54].

### Tiotropium bromide

The anticholinergic alkaloids contained in *Atropa belladonna*, *Datura stramonium* and *Hyosciamus niger* plants have been used as popular remedies to alleviate respiratory symptoms since ancient Egyptians times and the Middle Ages, when in Europe the deadly nightshade was used [76]. In the 19^th^ century atropine, a racemate of hyoscyamine [77], was isolated from nightshade, and became the progenitor of the modern anticholinergic compounds such as ipratropium. Since the '70s there was a renewed interest in anticholinergics as alternative options for the treatment of obstructive diseases, but ipratropium bromide, although demonstrated good clinical efficacy during acute asthma exacerbations [78], had a short duration of action and less bronchodilating effects compared with inhaled β2-agonists [78]. In the '90s tiotropium bromide was developed [79]. Tiotropium bromide is a synthetic quaternary ammonium anticholinergic agent derived from ipratropium bromide [80] (molecular formula C_19_H_22_BrNO_4_S_2_ [81]). Tiotropium demonstrated a high functional selectivity for specific M_R_, with a long duration of action, being approximately 10-fold more potent than ipratropium in binding M_R_ in vitro and in vivo studies [82]. Kinetic studies showed that tiotropium had a high selectivity for M_1R_ and M_3R_ and dissociated 100 times slower than ipratropium from M_1R_ (14.6 h vs 0.11 h) and M_3R_ (34.7 h vs 0.26 h) [83]. At the same time it had dissociation time from M_2R_ 10 times faster than from M_3R_, making it a selective functional antagonists of M_3R_ [79]. Further labeling and functional in vitro studies on human lungs confirmed its selectivity over M_2R_ [83]. Tiotropium is poorly absorbed from the gastro-intestinal tract and has very low systemic bioavailability [84]. After a single dose inhalation, peak plasma levels are reached after a maximum of 5 min, with a subsequent rapid decline in 1 h to very low levels (in the 2 pg/mL range) [85]. Tiotropium is than eliminated with a terminal half-life of 5 to 6 days, in a dose-dependent manner [85]. In vitro functional studies demonstrated that, although with a slower onset of action compared with ipratropium, the offset of tiotropium is very long compared with atropine, with a half-life of 300 min. These results justify the prolonged inhibitory effect of tiotropium in pharmacological in vivo studies. In asthmatic patients treated with tiotropium in doses from 10 to 80 μg, it demonstrated a protection against methacholine challenge for up to 48 h [86]; a prolonged and rapid onset of protection against methacholine challenge in patients with moderate airway hyper-responsiveness was later confirmed by Terzano and colleagues [87]. Analysis of pooled data from phase II and phase III RCTs in patients with asthma exposed to tiotropium delivered via Respimat® shows that tiotropium is rapidly absorbed rapidly, with maximum plasma concentrations 5 min post-inhalation. Compared with COPD patients, the peak plasma concentration in patients with asthma was approximately 52%, with no difference in exposure comparing the once daily and twice daily administration regimens. Age, allergic status, race, geographical region and smoking history did not appeared to influence the systemic exposure to tiotropium in patients with asthma [88]. In the numerous randomized clinical trials conducted in COPD [89], tiotropium showed an excellent and sustained bronchodilator effect [90], with proved efficacy in the reduction of static and dynamic volumes [91], in the reduction of the incidence of acute exacerbations [90], in the improvement of quality of life and symptoms [90], together with a good safety profile [91].

Approved for the long-term treatment of COPD since 2002 (2004 in the United States), tiotropium has been initially delivered via dry powder inhaler at the dosage of 18 μg qd; since 2014, tiotropium has been delivered also via Respimat® SoftMist™ inhaler technology, with lowered dosages (5 μg once daily). Following positive large efficacy and safety trials in asthma, in 2014 the European Medicines Agency has extended the indication of tiotropium Respimat® at a dose of 2.5 μg once daily (delivered in two inhalation of 1.25 μg each) to patients with asthma, which was also approved in more than 50 countries, including Japan. In September 2015 the Food and Drug Administration confirmed the same indication; the latter being extended for patients aged 6 and older since 16^th^ February 2017.

### Tiotropium non-bronchodilator effects

The immunopathologic response in asthma involves different inflammatory pathways and cells, mainly eosinophils, T-lymphocytes and macrophages which have a different role and are differently distributed in atopic and non atopic asthma [92, 93]. The inflammatory response is not limited to the migration of inflammatory cells in the epithelium of the airways, but is also characterized by the modulation and activity of the epithelial barrier per se, with the involvement of fibroblasts and smooth muscle cells [94]. As the majority of the involved cells express muscarinic receptors, the role of the neuronal and non-neuronal cholinergic system and thus of LAMA administration can go far beyond the modulation of the bronchomotor tone, enhancing the anti-inflammatory and bronchodilator effects of LABA [95]. We present here a brief overview of the evidences of tiotropium ancillary effects in vivo and in vitro.

#### Airway inflammation

Allergen inhalation, cigarette smoke and exogenous stresses like methacholine induce inflammatory M_3R_–mediated influx of eosinophils and neutrophils in the airways and are responsible for the production of numerous inflammatory mediator such as IL-8 and IL-6 [96]. However, the role of ACh in remodeling processes seems to be independent of the allergic inflammatory response and might be driven directly by bronchoconstriction, as demonstrated by the allergen-induced inflammation in knockout M_3R_ models [16]. Eosinophils are responsible for M_2R_ dysfunction in the lungs [16], while ACh is known to mediate the production of leukotriene-B4 (LTB4) and thus to lead neutrophil recruitment [97]. In animal models of allergic asthma, the protective effect of Tiotropium against airway hyper-responsiveness and airway inflammation was shown to be paralleled by a reduction in the eosinophil deposition in the airways [23, 98]. The authors speculate that in this case Tiotropium acted through non conventional pathways, non-neuronal ACh release from macrophages and epithelial cells or acting directly on M_3R_ present on eosinophils, although the existence of the latter is to be confirmed yet. A suppressive activity of tiotropium on the production of macrophages-derived chemotactic mediators has been previously described mostly in COPD models. In vivo [97] and in vitro [99] studies have demonstrated a reduction in LTB4-mediated inflammation promoted by tiotropium; a reduction in neutrophilic-mediated inflammation acting on the concentration of TNF-α, IL-6 and macrophage inflammatory proteins was also described in smoke induced neutrophilia [100] and in lipopolysaccharide-induced neutrophilia in a guinea pig model of COPD [101]. Although leukotriene-E4 inflammation represents a useful biomarker both for asthma control and treatment management, to date, studies addressing the potential role of tiotropium in the modulation of leukotriene-E4-induced inflammation in asthma are lacking. In a rhinovirus infection model of human tracheal cells, Turner and colleagues showed that tiotropium was able to reduce the viral RNA and cytokine levels in the supernatant fluid, reducing IL-6, IL-8 and IL-1β levels [102]. Eosinophilic recruitment was affected also by a concomitant administration of tiotropium and budesonide or ciclesonide in animal models of asthma [103, 104]. Recent evidences in guinea pig models of asthma demonstrated a synergistic action between olodaterol and tiotropium when administered together; in fact, tiotropium supported and enhanced the protection against allergen-induced hyper-responsiveness, fully inhibiting the early and late asthmatic reaction [105]. The latter mechanism may be explained by the evidence that during charbacol induced inflammation of human fibroblasts, tiotropium seem to sustain olodaterol in restoring c-AMP levels in airway fibroblasts, cooperating to decrease the inflammatory response [93].

#### Airway remodeling

Chronic inflammation in asthma drives the pathological structural remodeling of the airways. M_3R_ mediate airway smooth muscle thickening and extracellular matrix deposition [17]. Previous experiments with fibroblasts from patients with COPD and active smokers demonstrated an increased expression of ChAT, M_1R_ and M_3R_ compared with fibroblasts from healthy controls, associated with an increased proliferation following Ach administration [106], a phenomenon down regulated by tiotropium and mediated by M_R_, extracellular signal-regulated kinase (ERK) 1/2 and nuclear factor (NF) kappaB [106]. In cultured fibroblasts and myofibroblasts, tiotropium was shown to inhibit ACh-induced proliferation in a concentration-dependent fashion [107] and to inhibit the production of metallo-proteinases-2 (MMP-2) while sparing tissue inhibitors of MMP-1 and MMP-2 [108, 109]. Tiotropium also showed an anti-remodeling and anti-hypertrophy effect on SM in a guinea pig asthma model [26]. In mice chronic asthma models tiotropium significantly decreased smooth muscle thickening and peribronchial collagen deposition [69], with a reduction of Th2-mediated cytokines such as Il4, IL5 and IL13, TGF-β [69, 110]. The association of tiotropium and budesonide or ciclesonide demonstrated to have inhibitory effects on allergen-induced inflammation and remodeling in vivo, although not in acute conditions [103, 104].

#### Mucus production

An increased mucus production is associated with lower quality of life, less disease control and more exacerbations, especially in smoker patients with asthma [111]. In healthy airways, MUC5AC is the most common mucin gene normally produced, but its expression is markedly increased in asthma and is mediated by M_3R_ which induce mucus production in goblet cells [112]. Selective M_3R_ with tiotropium showed an inhibition of neutrophil elastase-induce goblet cell metaplasia and neutrophil-elastase induced MUC5AC production [113]. Concomitant administration of tiotropium and inhaled steroids like budesonide [103] and dexamethasone [98] showed to reduce allergen-induced mucus gland hypertrophy and mucus hyper secretion in mice models of allergic asthma. Although having an effect on mucus production, tiotropium was not demonstrated to alter the rheological properties of mucus. The reported reduction of mucociliary clearance induced by the inhalation of old antimuscarinic drugs and tertiary ammonium compounds such as atropine was not reported after inhalation of ipratropium or tiotropium [16].

### Cough reflex

Although the number of studies investigating the modulation of the cough reflex secondary to administration of antimuscarinic compounds is raising, the majority of them has been conducted in the 90’s and all showed a reduction in cough threshold with administration of ipratropium [114–118]. However, the disease models used, the stimuli (mechanical, chemical) and the scale employed to evaluate the cough response differ widely [119] and thus the interpretation of the results appears difficult. The evidences that studied the effects of tiotropium are limited and mostly in animal models. In subjects with upper viral respiratory tract infection tiotropium after the first dose and after 7 days, with no correlation with changes in pulmonary function, suggesting mechanisms other than the modulation of the bronchomotor tone [120]. Birrell and colleagues [121] showed that tiotropium, but not glycopyrronium, was able to modulate the cough reflex through the transient-potential vanilloid receptor type-1 (TRPV1) with mechanisms not related to its anticholinergic activity, but rather interacting with another binding site on the channel or by acting indirectly as a modulator of the TRPV1 channel [121]. Further insight was given by Mutolo and coworkers that suggested that the down-regulation of cough promoted by tiotropium implied also the involvement of acid-sensing ion channels and mechanoreceptors [122].

### Dose finding

#### Children (6–11 years)

A summary of phase II trials that investigated dose finding and safety of tiotropium in patients with uncontrolled asthma is reported in Table 2.

**Table 2:** Phase II dose finding and safety trials

Authors	Year	Main inclusion criteria	Age	Number	Treatment	Duration	Primary outcome	Secondary outcomes	Conclusions
Vogelberg C et al. [123]	2015	FEV_1_ 60–90%pred. Symptomatic with ACQ-7 > 1.5 and treatment with 200–400 μg of budesonide or eq. +/− LABA +/− LTRA	6–11	101	Add on tiotropium 1.25 μg, 2.5 μg, 5 μg or placebo to medium-dose ICS with or without LTRA	12 weeks	Peak FEV_1_(0–3 h)	1) Trough FEV_1_ 2) Trough FVC 2) FEV_1_AUC _(0–3h)_ 3) morning/evening PEF 4) ACQ-7 5) PAQLQ (S)	All doses were superior to placebo in all outcomes. No difference between doses.
Vogelberg C et al. [124]	2014	FEV_1_ 60–90%pred. Symptomatic with ACQ-7 > 1.5	12–17	105	Add on tiotropium 1.25 μg, 2.5 μg, 5 μg or placebo to medium-dose ICS with or without LTRA	4 weeks	Peak FEV_1_(0–3 h)	1) Trough FEV_1_ 2) FEV_1_AUC _(0–3h)_ 3) Morning/evening PEF 4) ACQ-7	The response of Tiotropium 5 μg dose is superior compared to placebo and greater than tiotropium 1.25 and 2.5 μg
Kerstjens HA et al. [125]	2011	Severe persistent asthma FEV_1_ < 80%pred. ACQ-7 > 1.5 and high dose ICS (≥800 μg budesonide or eq.) + LABA +/− teophylline, LTRA or OCS	18–75	100	Add on tiotropium 5 μg vs 10 μg vs placebo	24 weeks	Peak FEV_1_(0–3 h)	1) Trough FEV_1_ 2) Peak FVC 3) FVC AUC _(0–3h)_ 4) Trough FVC 5) FEV_1_AUC _(0–3h)_ 6) morning/evening PEF 7) Mini AQLQ 8) rescue medication use 9) asthma symptoms 10) symptom free days	Compared with placebo, both tiotropium doses were superior in all outcomes. There was no difference between doses.
Beeh KM et al. [128]	2014	FEV_1_ 60–90%pred. Symptomatic with ACQ-7 > 1.5 and medium dose ICS +/− LABA +/− SABA	18–75	141	Add on tiotropium 1.25 μg vs 2.5 μg vs 5 μg vs placebo	12 weeks	Peak FEV_1_(0–3 h)	1) Trough FEV_1_ 2) Peak FVC (0–3 h) 3) FVC AUC _(0–3h)_ 4) Trough FVC 5) FEV_1_AUC _(0–3h)_ 6) morning/evening PEF 7) ACQ 7	Compared with placebo, all tiotropium doses were superior in all outcomes. The largest response was obtained with 5 μg
Timmer W et al. [127]	2015	FEV_1_ 60–90%pred. Symptomatic with ACQ-7 > 1.5 and medium dose ICS +/− LABA +/− SABA	18–75	89	Add on tiotropium 5 μg OD vs 2.5 μg BID vs placebo	12 weeks	FEV_1_AUC _(0–24)_	1) Peak FEV_1_ (0–24 h) 2) Trough FEV_1_ 3) morning/evening PEF 4) ACQ-7	Both tiotropium doses are superior to placebo in all outcomes. No advantage of BID administration.
Ohta K et al. [129]	2015	FEV_1_ 60–90%pred. Symptomatic despite 400–800 μg budesonide or eq. +/− LABA	18–75	285	Add on tiotropium 2.5 μg, 5 μg or placebo to ICS +/− maintenance therapy	52 weeks	Long term safety	1) Trough FEV_1_ 2) Trough FVC 3) Trough PEF 4) ACQ-7	No difference in AEs rate between groups

Phase II dose finding and safety trials performed with tiotropium in patients with poorly controlled moderate and severe asthma. *N* number of patients randomized to treatment, *%pred* percent predicted, *eq*. equivalent, *AQLQ* Asthma Quality of Life Questionnaire, *PAQLQ* Pediatric Asthma Quality of Life Questionnaire, *OCS* oral corticosteroids. For other abbreviations please see text

Three different tiotropium Respimat® evening doses (1.25 μg, 2.5 μg and 5 μg) were tested in a phase II double-blind, placebo-controlled dose-ranging study in children with symptomatic asthma despite a maintenance therapy with medium-dose ICS with or without an antileukotriene (LTRA). Patients’ mean (standard deviation, SD) reversibility at screening was of 370 mL (171). After 4 weeks of treatment, the peak FEV_1_ in the first 3 h (0–3 h) post-dose period (primary endpoint) was improved by 87 mL (*p* = 0.0002), 104 mL (*p* < 0.0001) and 75 mL (*p* = 0.0011), for 5 μg, 2.5 μg and 1.25 μg respectively compared with placebo, with no statistical difference between doses. Similar significant improvements and no dose-dependent response were observed also for the mean trough FEV_1_ and the FEV_1_ area under the curve (AUC) between 0 and 3 h post-dose (0–3 h). All dosages improved the morning expiratory flow (PEF) compared to placebo, while the improvement in the evening PEF was limited to the highest dosage. Although not different compared to placebo, asthma control and quality of life were similarly improved, with a comparable and low rate of adverse events (AEs) [123].

### Adolescents (12–17 years)

A randomized, double-blind, placebo-controlled, incomplete crossover study evaluated once-daily tiotropium 5 μg, 2.5 μg and 1.25 μg delivered via Respimat® versus placebo in three 4-week treatment periods; primary efficacy end point was change in peak FEV_1_ (0–3 h). A significant improvement compared to placebo was found only for tiotropium 5 μg (113 mL, *p* = 0.0043). Again, the largest and only significant improvement compared to placebo for trough FEV_1_ was found for tiotropium 5 μg (adjusted mean: 151 mL; *p* < 0.0001). A slightly more frequent occurrence of AEs compared to other dosages and placebo was present in patients treated with tiotropium 5 μg, while serious AEs (4 in total, 1 in the tiotropium 5 μg group) were not considered related to the study treatment [124].

### Adults

Tiotropium 5 μg was demonstrated to be non-inferior to the dosage of 10 μg and superior to placebo in terms of lung function outcomes in a study involving patients with uncontrolled severe asthma despite treatment with high dose ICS/LABA [125]. The higher dose of tiotropium, however, was associated with a higher rate of dry mouth occurrence [125].

The pharmacokinetic profile of two regimens of inhaled tiotropium, 2.5 μg twice daily (TD) and 5 μg once daily (OD), was tested in a phase II randomized controlled two way crossed over trial involving asthmatic patients in maintenance therapy with budesonide 400–800 μg or equivalent [126]. The geometric mean of pre-dose plasma concentrations of tiotropium at steady state ranged between 1.43 pg/mL (5 μg OD) and 1.59 pg/mL (2.5 μg TD) [126], while the cumulative urinary excretion over 24 h was similar for both dosing regimens [126]. Accordingly, functional outcomes from Beeh et al. [126] and Timmer et al. [127] demonstrated no difference in FEV_1_AUC (0–24) for tiotropium 5 μg OD an d 2.5 μg TD, while both dose regimens lead to a significant improvement compared to placebo [126, 127].

Efficacy and safety of three doses (namely 1.25 μg to 2.5 μg and 5 μg) of once-daily tiotropium Respimat® were tested in a phase II, randomized, double-blind, placebo controlled, crossover study conducted in a population of moderate asthma patients treated with stable medium-dose ICS (400–800 μg budesonide or equivalent), alone or in a fixed-dose combination with a LABA. Lung function measured as peak FEV_1_ (0–3 h) improved for all doses of tiotropium Respimat® (*p* < 0.0001 for all doses) with a largest mean difference from placebo observed for tiotropium 5 μg (188 mL, 95% confidence interval, CI: 140, 236) [128].

#### Safety

Tiotropium long term safety as a primary outcome in asthma patients was specifically evaluated in a 52-weeks randomized controlled study in which tiotropium 5 μg, 2.5 μg and placebo were administered in symptomatic Japanese patients as add-on therapy to ICS/LABA. The most common AEs reported were nasopharyngitis (48.2%, 44.7%, 42.1%), asthma (28.9%, 29.8%, 38.6%), decreased PEF (15.8%, 7.9%, 21.1%), bronchitis (9.6%, 13.2%, 7.0%), pharyngitis (7.9%, 13.2%, 3.5%) and gastroenteritis (10.5%, 3.5%, 5.3%) for 5 μg, 2.5 μg and placebo respectively. The rate of serious adverse effects was slow and not different between the study groups [129]. In the majority of phase II and III trials that evaluated tiotropium in asthma, patients with narrow angle glaucoma and symptomatic prostatic hypertrophy were excluded; nonetheless, considering the importance of such comorbidities in late onset asthma, the administration of tiotropium was not related to an increased frequency of AEs involving the eye, reproductive and urinary tract apparatuses. In the study conducted by Ohta et al. [129], the frequency of AEs related to eye disorders was usually very low, and lower in the tiotropium treated groups compared to placebo (5.3 and 6.1 vs 8.8% for patients treated with tiotropium 5 μg, 2.5 μg and placebo) [129]. In the same trial, cystitis was reported respectively by 4.4% (tiotropium 5 μg), 2.6% (tiotropium 2.5 μg) and 1.8% (placebo) of patients. A recent pooled analysis of randomized, double blind parallel group phase II and III trials [130] investigated the safety profile of tiotropium 5 μg and 2.5 μg, compared to placebo. Out of 3,474 patients analyzed, 2,157 received tiotropium. The overall percentage of patients reporting AEs was not different between groups, being 60.8% vs 62.5% for tiotropium 5 μg and placebo 5 μg and 57.1% vs 55.1 for tiotropium 2.5 μg and placebo 2.5 μg. The most common AEs were represented by a reduction in PEF and nasopharyngitis. Adverse cardiac events were comparable between active treatments and placebo. AEs typically associated with use of LAMAs such as dry mouth was very low, ranging from 0.4% for tiotropium 2.5 μg to 1.0% for tiotropium 5 μg. Serious AEs were similar between groups, albeit slightly higher for 5 μg dose compared to 2.5 μg (4.0% vs 2.0%, respectively) [130].

#### Efficacy

Following proof of concept studies, the efficacy and safety of tiotropium delivered via Respimat® was investigated in a large scale clinical trial program (the UniTinA-asthma®) and in many independent studies, which included children, adolescents and adult patients. We summarized the main findings of the RCTs published so far dividing them by patients’ age (Table 3). If not differently stated, all tiotropium doses are meant to be delivered by Respimat® SofMist™ inhaler.

**Table 3:** Phase III RCTs that evaluated efficacy and safety of tiotropium in asthma

Authors	Year	Main inclusion criteria	Age	Number	Treatment	Duration	Primary outcome	Secondary outcomes	Conclusions
Children and adolescents									
Szefler SJ et al. [133]	2017	FEV_1_ 60–90%pred ACQ-7 > 1.5 and high dose ICS + 1 controller therapy or medium dose ICS + 2 controller therapies	6–11	392	Add on tiotropium 5 μg, 2.5 μg or placebo to chronic medium dose ICS (200–400 μg budesonide or eq.) + 2 controller or high dose ICS (≥400 μg) plus one controller	12 weeks	Peak FEV_1_ (0–3 h)	1) Trough FEV_1_ 2) Peak FVC (0–3 h) 3) ACQ–IA score and responder rate 4) trough FVC 5) FEV_1_AUC _(0–3h)_ 6) rescue medication use 7) time to first exacerbation 8) time to first severe exacerbation 9) ACQ-6 and ACQ-7 10) FEF_25–75_ 11) weekly evening PEF 12) Tolerability	Primary and key secondary outcomes were significantly improved only for tiotropium 5 μg. Peak FVC (0–3 h) and trough FVC did not reach significance for any tiotropium dose.
Huang J et al. [134]	2016	Moderate persistent asthma	6–14	80	125 μg fluticasone propionate aerosol TD + placebo OD vs 125 μg fluticasone propionate aerosol TD + tiotropium 18 μg dry-powder OD	12 weeks	(not clearly stated)	1) FEV1, FVC and PEF at week 12. 2) Asthma exacerbation 3) Rescue medication use 4) Night time symptoms 5) Tolerability	Tiotropium 18 as add-on to maintenance therapy significantly improved lung function compared to maintenance therapy alone.
Hamelmann E et al. [131]	2016	FEV_1_ 60–90%pred ACQ-7 > 1.5 and chronic treatment with ICS (200–800 μg for 12–14 years; 400–800 μg for 15–17 years) +/−LABA +/− LTRA	12–17	376	Add on tiotropium 5 μg, 2.5 μg or placebo to maintenance therapy, with ICS +/− LTRA (LABA not permitted) + open label SABA as rescue medication	48 weeks	Peak FEV_1_ (0–3 h)	1) Trough FEV_1_ 2) Peak FVC (0–3 h) 3) FVC AUC _(0–3h)_ 4) Trough FVC 5) FEV_1_AUC _(0–3h)_ 6) time to first exacerbation 7) time to first severe exacerbation 8) ACQ-7 and ACQ-6 9) AQLQ (S) score and responder rate 10) Tolerability	All functional outcomes were significantly improved compared to placebo for all tiotropium doses. Greatest overall benefit was found for tiotropium 5 μg. A trend towards improvements was present for ACQ-7
Hamelmann E et al. [132]	2016	ACQ-7 > 1.5 and high dose ICS + 1 controller therapy or medium dose ICS + 2 controller therapies	12–17	388	Add on tiotropium 5 μg, 2.5 μg or placebo to chronic ICS plus one or more controller therapies.	12 weeks	Peak FEV_1_ (0–3 h)	1) Trough FEV_1_ 2) Peak FVC (0–3 h) 3) FVC AUC _(0–3h)_ 4) trough FVC 5) FEV_1_AUC _(0–3h)_ 6) rescue medication use 7) time to first exacerbation 8) time to first severe exacerbation 9) ACQ-6 and ACQ-7 10) FEF_25–75_ 11) evening and morning PEF 12) Tolerability	Primary and secondary endpoint not met. Numerical greater response with tiotropium 5 μg compared to placebo.
Adults									
Peters SP et al. [137] “TALC study”	2010	Symptomatic despite 160 μg daily beclomethasone with FEV_1_ < 70%pred	≥18	174	Tiotropium 18 μg + placebo vs beclomethasone 320 μg + placebo vs salmeterol 50 TD + beclomethasone 160 μg + placebo	14 weeks – 3 period	Morning PEF	1) Trough FEV_1_ 2) asthma control days 3) rescue medication use 4) asthma symptoms 5) exacerbations 6) use of health care service 7) inflammatory biomarkers 8) ACQ 9) Tolerability	Tiotropium 18 μg is superior to doubling the ICS dose and non-inferior to salmeterol in patients with uncontrolled asthma
Wang K et al. [138]	2015	Moderate asthma. FEV_1_ 60–80%pred, daily use of SABA, PEF and FEV_1_variability of >30%. ACT 12–20	≥18	94	Add on therapy with tiotropium 18 μg, LTRA or double dose ICS on salmeterol/fluticasone dry-powdre 50/250 μg TD	16 weeks	Asthma control in terms of FeNO; daily PEF variability and ACT score	Not clearly stated	Tiotropium non inferior to doubling doses of ICS. Best response obtained with double dose ICS/LABA but higher risk of pneumonia and RTI.
Kerstjens HA et al. [139] “PrimoTinA-asthma 1 & PrimoTinA-asthma 2“	2012	Uncontrolled asthma defined with ACQ-7 > 1.5, FEV_1_ ≤ 80%pred and/or FVC ≤ 70%pred despite chronic treatment with ≥800 μg budesonide + LABA	18–75	912 (456 per study)	Tiotropium 5 μg add on therapy or matching placebo. Teophylline, OCT and LTRA were permitted if part of maintenance therapy along with LABA/ICS.	Two replicate 48 weeks	1) Peak FEV_1_ (0–3 h) 2) Trough FEV_1_ (24 weeks). 3) Time to first exacerbation (48 weeks)	At each visit: 1) Trough FEV_1_ 2) Peak FEV_1_ 4) trough FVC 5) Peak FVC 6) FEV_1_AUC _(0–3h)_ 7) FVC AUC _(0–3h)_ 8) time to first exacerbation 9) morning and evening PEF 10) asthma symptoms 11) ACQ-7 and AQLQ 12) Tolerability	Add on treatment with tiotropium to ICS/LABA sustained bronchodilation over 24 h, reduces severe exacerbations and episodes of worsening of disease. Improvements in asthma control scores and other secondary outcomes were not met.
Kerstjens HA et al. [141] “MezzoTinA-asthma 1 & MezzoTinA-asthma 2“	2015	Uncontrolled asthma defined with ACQ-7 > 1.5, FEV1 60–90%pred despite chronic treatment with 400–800 μg budesonide or eq. +/− LABA or SABA	18–75	1972 (998 & 974 per study)	Tiotropium 5 μg, 2.5 μg, salmeterol 50 μg TD or placebo as add on therapy to 400–800 μg of budesonide or eq.	Two replicate 24 weeks	1) Peak FEV1 (0–3 h) 2) Trough FEV1 (24 weeks). 3) ACQ-7 responder rate	1) trough FVC 2) Peak FVC 3) morning weekly PEF 4) ACQ-7 5) time to first exacerbation 6) Tolerability	Add on treatment with tiotropium significantly improves lung function and asthma control compared with placebo, and has similar efficacy and tolerability to salmeterol
Paggiaro P et al. [142]	2016	Uncontrolled asthma defined with ACQ-7 > 1.5, FEV1 60–90%pred despite chronic treatment with 200–400 μg budesonide or eq.	18–75	464	Tiotropium 5 μg or tiotropium 2.5 μg or placebo as add on treatment to chronic low to medium ICS.	12 week	Peak FEV_1_ (0–3 h)	1) Trough FEV_1_ 2) FEV_1_AUC _(0–3h)_ 3) Use of rescue medication 4) ACQ-7 5) morning and evening PEF 6) Safety	Both doses of tiotropium were significantly superior to placebo for every lung function outcome. No effect size retrieved. No difference in reduction of ACQ-7 score between active and placebo groups.

Phase III RCTs that evaluated efficacy and safety of different doses of tiotropium in patients with moderate to severe uncontrolled asthma. RCTs are divided according to the study sample age. *N*  number of patients randomized to treatment, *ACQ-IA* Interviewer-Administered version of the Asthma Control Questionnaire, *RTI* respiratory Tract Infections, *FeNO* Exhaled Fraction of Nitric Oxide, *%pred* percent predicted, *eq.* equivalent. For other abbreviations please see text. In all trials adverse effects were not different between groups if not otherwise reported. For other abbreviations, please see text

### Children and adolescents

The efficacy of tiotropium was recently evaluated in patients <18 years old in four large, randomized double blind placebo controlled studies. A phase III, parallel-group 52 weeks RCT compared tiotropium 5 μg (administered by 2 inhalations of 2.5 μg), tiotropium 2.5 μg and placebo Respimat® given in the evening in adolescent patients with moderate symptomatic asthma. Symptomatic asthma was defined as an ACQ-7 mean score of at least 1.5. Patients had to be on maintenance therapy with ICSs, which was permitted during the study, with or without a LABA or a LTRA. Peak FEV_1_ (0–3 h) after 24 weeks was the primary outcome. 376 patients completed the study. Peak FEV_1_ (0–3 h) was significantly and similarly improved for both tiotropium doses (174 mL [95% CI, 76–272 mL] for tiotropium 5 μg and 134 mL [95% CI,34–234 mL] for tiotropium 2.5 μg) compared with placebo. A rapid improvement of the forced expiratory flow (FEF) between 25% and 75% of FVC (FEF_25–75_) compared with placebo was also present beginning from 10 min post-dose for tiotropium 5 μg and 1 h post dose with the 2.5 μg dose. Trough FEV_1_ was significantly improved compared to placebo only for tiotropium 5 μg: adjusted mean (standard error) 400 mL (41), +117 mL (54) vs placebo, *p* = 0.03 [131]. The same outcomes have been investigated by Hamelmann and coworkers [132] in adolescent patients with severe symptomatic asthma (ACQ-7 mean score of ≥1.5) despite medium to high doses of ICS (from 400 μg to 1600 μg of budesonide or equivalent in patients aged 15–17 and >400 μg in those aged 12–14 years) and one or more controller therapies (LABA and/or LTRA). Peak FEV_1_ (0–3 h) compared with placebo was significantly improved only with tiotropium 2.5 μg (111 mL; *p* = 0.046), while all other outcomes, although showing a trend towards improvement, were not met [132]. The first and only phase III trial to assess the efficacy and safety of once-daily tiotropium add-on therapy in children with severe symptomatic asthma was published by Szefler SJ and colleagues [133]. Patients had to be symptomatic despite a maintenance therapy with medium ICS with two or more controller medications or high dose ICS with one or more controller medications and were randomized to receive tiotropium 5 μg, 2.5 μg or placebo for 12 weeks. Compared with placebo, only add-on tiotropium 5 μg significantly improved peak FEV_1_ (0–3) (139 mL; 95% CI, 75–203), and trough FEV_1_ (87 mL; 95% CI, 19–154) with a good safety profile [133].

The efficacy of tiotropium 18 μg administered by dry powder inhaler was tested in children and adolescents with moderate persistent asthma and compared with fluticasone 125 μg via aerosol twice daily. After 12 weeks of treatment, the tiotropium/fluticasone group experienced significant improvements in FEV_1_, FVC and PEF and a reduced usage of SABA on demand therapy and night-time symptoms compared with fluticasone alone [134].

### Adults

Initial proof of concept studies were conducted between late '90s and 2000 in adult asthmatic patients to prove tiotropium efficacy in protecting from methacholine-induced bronchoconstriction. O’Connor and colleagues [86], using doses of 10 μg, 40 μg and 80 μg, demonstrated that tiotropium was able to produce and maintain for 48 h a dose-dependent protection against methacholine challenge at 2 h [mean (standard error): 5.0 (1.1); 7.1 (0.5) and 7.9 (0.7) doubling doses], despite a mild increase in FEV_1_ that ranged between 5.5% and 11.1% from baseline [86]. Later on, Terzano and coworkers showed that tiotropium at a dose of 18 μg delivered via HandiHaler® had a protective effect against methacholine-induced bronchoconstriction in asthma patients with airway hyper-responsiveness. In fact, compared with placebo, patients treated with tiotropium did not reach the provocative dose causing a 20% decrease in basal FEV_1_ [87]. Another double blind, randomized, placebo controlled crossover study investigated the possibility to introduce tiotropium in order to step-down the ICS doses in severe asthma patients (mean FEV_1_51% predicted). While in patients treated with fluticasone 1000 μg + LABA lung function improvements were limited to PEF and airway resistances, only patients treated with tiotropium 18 μg, fluticasone 500 μg + LABA experienced also significant improvements in FEV_1_ (+170 mL) and FVC (+240 mL) which were associated also to a reduction in exhaled nitric oxide by 2.86 ppb compared to placebo [135]. Iwamoto and colleagues demonstrated also that improvements in airway obstruction following administration of tiotropium (dose not reported) in patients with severe asthma were positively correlated with neutrophil inflammation assessed by induced sputum, suggesting that tiotropium would be more effective in asthma patients with a non-eosinophilic phenotype [136].

The first large trial investigating the possibility to introduce tiotropium in the regular treatment in patients with uncontrolled asthma was the Tiotropium Bromide as an Alternative to Increased Inhaled Glucocorticoid in Patients Inadequately Controlled on a Lower Dose of Inhaled Corticosteroid (TALC) study [137]. In 210 patients with moderate to severe uncontrolled asthma with low doses of beclomethasone (80 μg daily), the investigators evaluated the addition of tiotropium 18 μg to beclomethasone 80 μg daily as compared with doubling the dose of the ICS (primary superiority comparison) or with the addition of salmeterol to beclomethasone 80 μg (secondary non-inferiority comparison). The addition of tiotropium to low-dose ICS resulted in significant improvements in all lung function and clinical outcomes compared to doubling the ICS dosage. Compared to the latter group, the association of tiotropium improved both morning and evening PEF were improved with tiotropium (+25.8 l/min; 95% CI, 14.4–37.1;*p* < 0.001 and +35.3 l/min; 95% CI, 24.6 – 46.0; *p* < 0.001, respectively), the pre-dose FEV_1_ (+100 mL, *p* = 0.004), the proportion of asthma-control days, score for daily symptoms, and the ACQ-7 (−0.18 points; *p* = 0.02). Moreover, tiotropium add-on therapy resulted to be non inferior to the LABA/ICS association in all outcomes [137]. Confirmatory results came from a single-center study in which treatment with tiotropium + LABA + low dose ICS was non inferior in functional outcomes and nitric oxide reduction to LABA + double dose ICS in patients with uncontrolled asthma [138].

Subsequently, two replicate, randomized, placebo-controlled 48-week period trials (PrimoTinA-asthma 1 and PrimoTinA-asthma 2) were designed to specifically assess if tiotropium 5 μg add on therapy to high dose ICS + LABA compared to add-on placebo was effective in improving disease control both in terms of lung function [peak FEV_1_ (0–3 h) and through FEV_1_] and of time to first exacerbation in patients poorly controlled with maintenance therapy [139]. Patients had to have a FEV_1_ < 80% predicted and a history of at least 1 severe asthma exacerbation in the previous year. Change in peak FEV_1_ (0–3 h) from baseline was significantly greater with tiotropium than with placebo in both trials (vs placebo, mean ± SE: 86 ± 34 mL and 154 ± 32 mL), and this was true also for the trough FEV_1_ (vs placebo: 88 ± 31 mL and 111 ± 30 mL). Time to the first severe exacerbation was increased in patients treated with add-on tiotropium (282 days vs. 226 days), with an overall reduction of 21% in the risk of a severe exacerbation (hazard ratio: 0.79; *p* = 0.03) [139]. The response to treatment with tiotropium was shown to be independent of baseline characteristics including gender, age, body mass index, disease duration, age at asthma onset, FEV_1_% predicted at screening and reversibility [140].

The non inferiority of tiotropium compared to LABA as add-on therapy to ICS in patients with moderate asthma was investigated in two 24-week, replicate, randomized, double-blind, placebo-controlled, parallel-group, active comparator trials (MezzoTinA-asthma 1 and MezzoTinA-asthma 2) [141]. These two parallel trials randomized a total of 2,103 patients to receive tiotropium 5 μg OD, tiotropium 2.5 μg OD, salmeterol 50 μg BID or placebo on top of maintenance therapy with medium dose budesonide (400–800 μg). Again, the two functional co-primary end-points were peak FEV_1_ (0–3 h) and through FEV_1_ at the end of the 24-week treatment period, additionally, the study assessed the proportion of responders by means of the ACQ-7, i.e. patients that increased the ACQ-7 score more than 0.5. Pooled data of the 1,972 patients that completed the studies showed significant improvements for both doses of tiotropium and salmeterol compared to placebo in all functional outcomes. Compared to placebo, both doses of tiotropium and salmeterol significantly improved peak FEV_1_ (0–3 h) and trough FEV_1_, although results were numerically higher for tiotropium 2.5 μg. There were more ACQ-7 responders in the tiotropium 5 μg (OR 1.32; 95% CI: 1.02–1.71; *P* = 0.035), 2.5 μg (1.33; 95% CI: 1.03–1.72; *P* = 0.031) and the salmeterol group (1.46, 95% CI: 1.13–1.89; *p* = 0.0039), compared to placebo. Although median time to first severe exacerbation could not be calculated because less than 50% of patients in each treatment group had one or more severe exacerbations, a statistically significant reduction in risk of first severe exacerbation was reported for tiotropium 2.5 μg and of first asthma worsening for tiotropium 2.5 μg and salmeterol [141]. A recent phase III RCT compared tiotropium 2.5 μg and 5 μg OD versus placebo as add-on therapy to maintenance treatment with ICS in patients with moderate symptomatic asthma with a FEV_1_ of 60 to 90% predicted. Data from 464 patients were analyzed. After 12 weeks of treatment, both dose regimens showed superiority over placebo in terms of through FEV_1_and peak FEV_1_ (0–3 h); however, numerically better results for peak FEV_1_ (0–3 h) and FEV_1AUC_ (0–3 h) were found for tiotropium 2.5 μg compared to 5 μg [adjusted mean (SE): 293 mL (26) vs 262 mL (26) and 198 mL (24) vs 174 mL (25), respectively]. The frequency of AEs was not different across the treatment regimens [142].

These large studies demonstrated the efficacy of tiotropium as add-on therapy both to moderate-to-high dose of ICS and to ICS/LABA maintenance therapy in terms of lung function, risk of exacerbation and disease worsening, goals that were achieved with a safety profile similar to LABA comparators and placebo. Further *post hoc* analyses of pooled data from MezzoTinA-asthma 1, MezzoTinA-asthma 2, PrimoTinA-asthma 1 and PrimoTinA-asthma 2 suggested that the efficacy of tiotropium was independent of underlying allergic/eosinophilic inflammation and thus of the T-helper 2 asthma phenotype, as outcomes were reached in patients with a broad range of IgE and eosinophil values [143].

The Arg16/Arg16 β2-adrenergic receptors polymorphism represents a common finding both in African Americans (20%) and Caucasians (15%) [59] and is associated with a blunted sensitivity to the maintenance asthma treatment with SABAs thus justifying a worse disease control with long term treatment with ICS/LABA FDC both in adults [144] and in children [145]. This assumption led to investigate the predictors of response to therapy by means of a pharmacogenetic approach. Sequencing of eleven different nucleotide polymorphisms was performed in 138 asthmatic not controlled with their maintenance therapy in which tiotropium 18 μg was added once daily. The positive response to tiotropium in terms of lung function was found in 33% of patients and was predicted by the Arg16Gly polymorphism [146]. When a cohort of asthmatic patients homozygous for the Arg16 β2 adrenergic receptor polymorphism were prospectively studied, tiotropium demonstrated to be non inferior to salmeterol in improving the morning PEF (mean ± SD; −3.9 ± 4.87 L/min for tiotropium and −3.2 ± 4.64 L/min for salmeterol) [147]. Given the proportion of African-American patients with allelic variations associated with poor asthma control, a study specifically powered and designed to assess the risk of exacerbations in this particular population was set in 2011. The Blacks and Exacerbations on LABA vs Tiotropium (BELT study) [148] was a parallel-group, randomized pragmatic trial that enrolled black adults with asthma from primary care and specialty practices in the United States. Participants were enrolled if on step 3 or step 4 combination ICS and LABA therapy, and were randomized to receive a LABA (salmeterol or formoterol) or tiotropium in addition to their chronic dose of ICS. The primary outcome was time to asthma exacerbation, secondary outcomes included patient-reported outcomes, lung function (FEV_1_ changes), rescue medication use and AEs. The investigators found no difference between the two treatment regimens in terms of primary and secondary outcomes. Neither of the Arg16Gly β2-adrenergic receptors alleles was associated with differences in the effects of tiotropium + ICS vs LABA + ICS [148].

Initial validations of these RCTs in real life settings can be found in two recent retrospective studies. An analysis of 64 patients with poor disease control despite treatment with high dose ICS/LABA showed that the introduction of tiotropium as add on therapy improved asthma control in 42.2% of cases, decreasing the number of emergency department visits and hospitalizations in 46.9% and 50.0% of cases, respectively [149]. A larger cohort involving 2,042 outpatients from United Kingdom compared the number of exacerbations (emergency visits, hospitalizations and oral corticosteroids use) and acute asthma events (antibiotic use for lower respiratory tract infections) in the year before and in the year following the prescription of tiotropium. Patients experienced an overall significant decrease in exacerbations and acute asthma events by 10% and 11%; however, there was a significant increase in the as needed usage of SABA from a median (IQR) of 274 (110–548) to 329 (110–603) μg/day [150].

## Conclusions

**Fig. 1:**
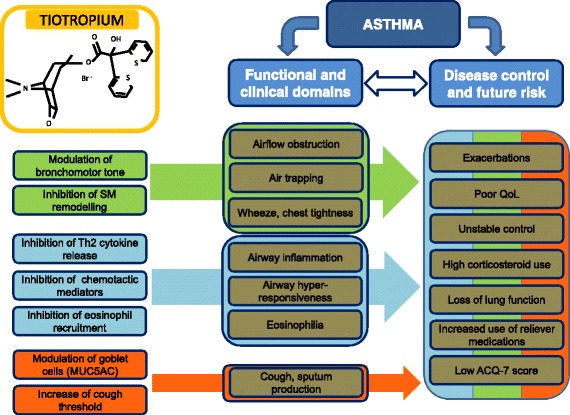
Possible role of tiotropium bromide in the management of asthma. The colour code refers to the functional and clinical characteristics that tiotropium should be able to modify according to its pharmacological properties. If the effect on functional and clinical asthma domains is effected prevalently by specific tiotropium properties, the effect on asthma control and future risk might be modulated by the concomitant action of different characteristics taken together. Th2, T helper-2 lymphocytes; SM, smooth muscle cells; MUC5AC, mucin-5 subtype AC gene; QoL, Quality of Life; ACQ-7, Asthma Control Questionnaire

Asthma control represents the main goal of numerous respiratory societies worldwide and a lot of energy has been spent so far trying to change quality of life of patients with uncontrolled asthma. Beside patient dependent factors, advances in maintenance therapy represent one of the cornerstones of risk management and disease control. Following its proven efficacy and safety in patients with COPD, a large body of evidence is now available for the use of tiotropium also in patients with poor controlled asthma. Large RCTs performed with patients with moderate to severe asthma have demonstrated its efficacy in improving lung function, decreasing risk of exacerbation and slowing the worsening of disease as add-on therapy, and its non inferiority compared to LABAs along the ICS maintenance treatment. In view of the ancillary and the synergistic effects provided by the cross-talk between β2 and muscarinic receptors, tiotropium can represent a beneficial alternative in the therapeutic management of poor controlled asthma (Fig. 1). Tiotropium safety profile and the broad spectrum of efficacy demonstrated in different disease phenotypes allows its use also in the pediatric and allergic population. More studies in human models of asthma are needed to explore its in-vivo anti-inflammatory effects, its impact on lung volumes and airway resistances and thus its role as disease modifier in the natural history of asthma.

## Abbreviations

95%CI: 95% Confidence interval
ACh: Acetylcholine
ACQ-7: Asthma control questionnaire
AEs: Adverse events
AUC: Area under the curve
ChAT: Choline acetyl-transferase
COPD: Chronic obstructive pulmonary disease
FDC: Fixed dose combination
FEV_1_: Forced expiratory volume in one second
FEV_1_ (0–3 h): Peak forced expiratory volume in one second in the first 3 h post-administration
FVC: Forced vital capacity
ICS: Inhaled corticosteroids
IQR: Inter quartile range
LABA: Long acting β2-agonists
LAMA: Long acting muscarinic antagonists
LTRA: Antileukotriene
MR: Muscarinic receptors
MUC5AC: Mucin-5 subtype AC gene
OD: Once daily
PEF: Peak expiratory flow
QoL: Quality of life
RCT: Randomized controlled trial
SABA: Short acting β2-agonists
SAMA: Short acting muscarinic antagonists
SD: Standard deviation
SE: Standard error
SM: Smooth muscle
TD: Twice daily
Th2: T lymphocytes type 2
