# Erratum to: The evidence on tiotropium bromide in asthma: from the rationale to the bedside

**DOI:** 10.1186/s40248-017-0099-y

**Published:** 2017-06-01

**Authors:** Dejan Radovanovic, Pierachille Santus, Francesco Blasi, Marco Mantero

**Affiliations:** 1 Department of Biomedical and Clinical Sciences (DIBIC), University of Milan, Pulmonary Unit, Ospedale L. Sacco, ASST Fatebenefratelli-Sacco, Milan, Italy; 2 Department of Pathophysiology and Transplantation, University of Milan, Cardio-thoracic unit and Adult Cystic Fibrosis Center, Fondazione IRCCS Ca’ Granda Ospedale Maggiore Policlinico, Milan, Italy

## Erratum

After publication of the Review article [1] it was brought to our attention that the sentence at the bottom of the section entitled Tiotropium bromide (pag 5) must be corrected as follows: “Following positive large efficacy and safety trials in asthma, in 2014 the European Medicines Agency has extended the indication of tiotropium Respimat® at a dose of 5 μg once (delivered in two inhalation of 2.5 μg each) to patients with asthma, which was also approved in more than 50 countries, including Japan. In September 2015 the Food and Drug Administration confirmed the same indication, but with a dosage of 2.5 μg once daily (delivered in two inhalation of 1.25 μg each); the latter being extended for patients aged 6 and older since 16th February 2017.”
